# When nerve turns to bone: a case report of neuritis ossificans in a total brachial plexus injury

**DOI:** 10.1097/RC9.0000000000000424

**Published:** 2026-03-26

**Authors:** Dina Aprilya, Oryza Satria, Jonathan Junius Sibarani

**Affiliations:** Orthopedic and Traumatology Department, Fatmawati General Hospital, Jakarta, Indonesia

**Keywords:** brachial plexus injury, case report, heterotopic ossification, neuritis ossificans

## Abstract

**Introduction and importance:**

Neuritis ossificans is a form of heterotopic ossification in which extra-skeletal bone formation occurs in nerves. Neuritis ossificans of the brachial plexus is a rare disorder, with only two cases reported in the literature to date.

**Case presentation:**

We report a case of neuritis ossificans of the brachial plexus in a 17-year-old patient presenting with flaccid paralysis and numbness of her left arm that occurred after an initial trauma.

**Clinical discussion:**

We performed surgical excision and nerve transfer of the brachial plexus. The tissue appeared as a bony envelope encasing the nerve. Histopathological examination confirmed it as a cartilaginous tissue. The patient’s symptoms gradually improved during follow-up.

**Conclusion:**

Neuritis ossificans of the brachial plexus is a rare disease. Surgical excision of the calcification and nerve transfer yielded a satisfactory outcome.

## Introduction

Neuritis ossificans is a rare disorder of extraskeletal bone formation within nerves^[^1,2^]^. Neuritis ossificans involving the brachial plexus is particularly rare, with only two cases reported in the literature. In these two reports, management included surgical excision and pharmacological therapy^[^3,4^]^. Here, we present the first reported case of neuritis ossificans of the brachial plexus following a total brachial plexus injury after trauma, treated with surgical excision and nerve transfer. This case report is reported in accordance to the SCARE 2025 Guideline[5].

## Case presentation

We report a case of a 17-year-old female patient who presented with paralysis of her left arm after falling from a slide with her left shoulder impacting the ground first 3 months ago. After the initial fall, she could not move her arm. She also reported numbness and pain in her arm, which were worst upon waking from sleep. Because of her symptoms, she was brought to a traditional bonesetter, but her symptoms did not improve. Then she was brought to the regional hospital. There, she was diagnosed with a brachial plexus injury and referred for further diagnostic workup and treatment.

HIGHLIGHTS

- Neuritis ossificans may occur in a total brachial plexus injury.
- The brachial plexus is a rare site of neuritis ossificans.
- Surgical excision of ossification followed by nerve transfer yielded a satisfactory outcome.

On physical examination in the orthopedic outpatient clinic, her left arm appeared smaller than the right, with flaccid paralysis. Neurological examination showed the following muscle strength in the left arm: shoulder shrug 5/5, shoulder abduction 0/5, elbow flexion 0/5, elbow extension 0/5, wrist flexion 0/5, wrist extension 0/5, finger flexion 0/5, and finger extension 0/5. The sensory function of her left arm was absent, physiological reflexes were absent, and the Hoffman–Tromner pathological reflex was negative. These findings suggested a total left brachial plexus injury.

**Figure 1.**
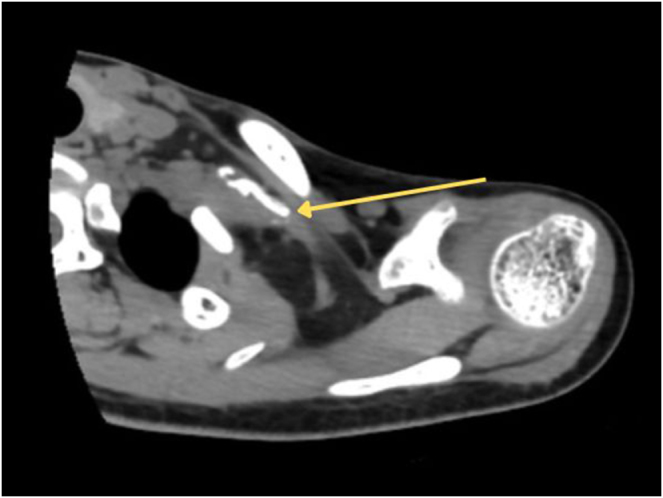
Preoperative axial shoulder CT scan showing the *double clavicle* sign, an ossified mass of brachial plexus (yellow arrow) located between the clavicle and the first rib.

Further diagnostic workup was done on the patient. The preoperative axial shoulder CT scan demonstrated an ossified mass located posterior to the clavicle. We propose the term *double clavicle* sign to describe this finding, defined as a bone-density mass representing ossification of the brachial plexus situated between the clavicle and the first rib (Fig. 1). The X-ray showed no signs of fracture. The electromyography examination showed abnormal results in the left deltoid, extensor digitorum communis, abductor digiti minimi, rhomboid major, and supraspinatus muscles. Nerve conduction velocity studies demonstrated absent motoric conduction response of the median, ulnar, radial, axillary, and musculocutaneous nerves and showed normal sensory conduction results in the ulnar nerve, reduced peak amplitude in the median nerve, and reduced peak amplitude in the radial nerve. The electromyography and nerve conduction velocity study concluded a total functional lesion of the left C5 nerve root and preganglionic denervation of the rest of the left brachial plexus. The patient was diagnosed with a total left brachial plexus injury and scheduled for a two-stage surgery to restore nerve function.

**Figure 2.**
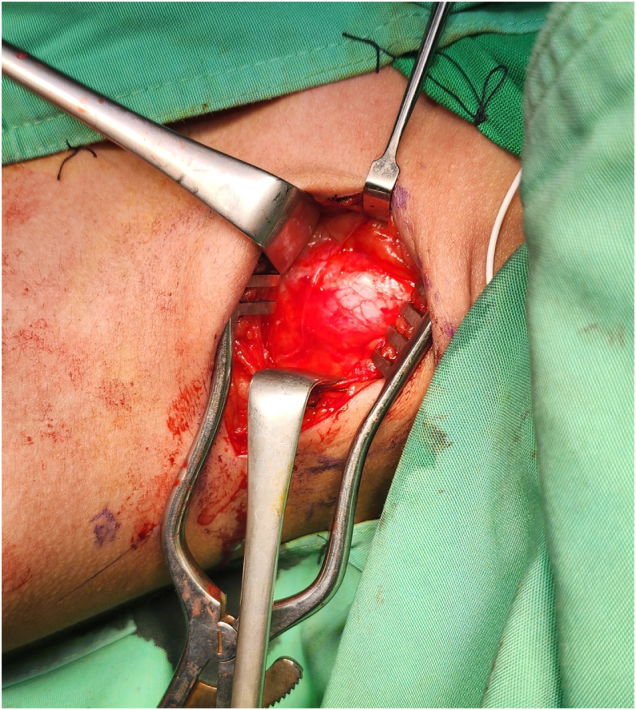
Bony envelope encasing the brachial plexus.

**Figure 3.**
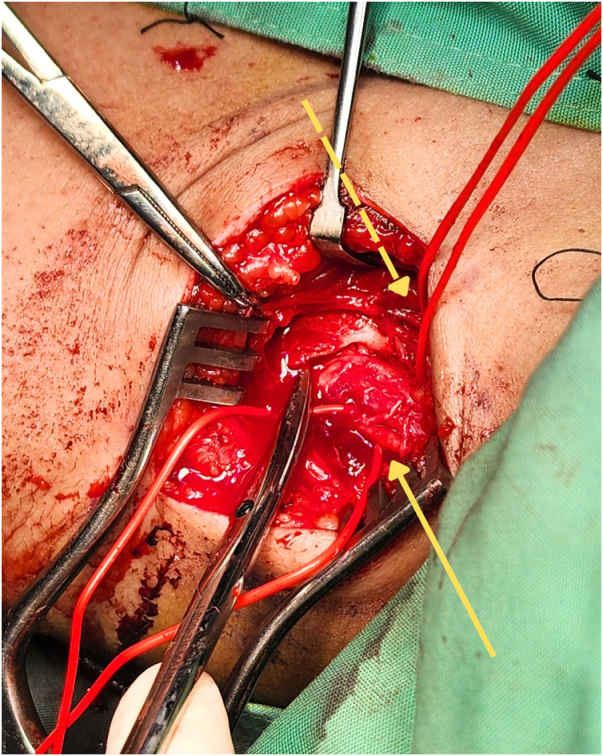
Bony envelope encasing the suprascapular nerve (solid arrow) and the middle trunk of the brachial plexus (dashed arrow) was easily detachable.

**Figure 4.**
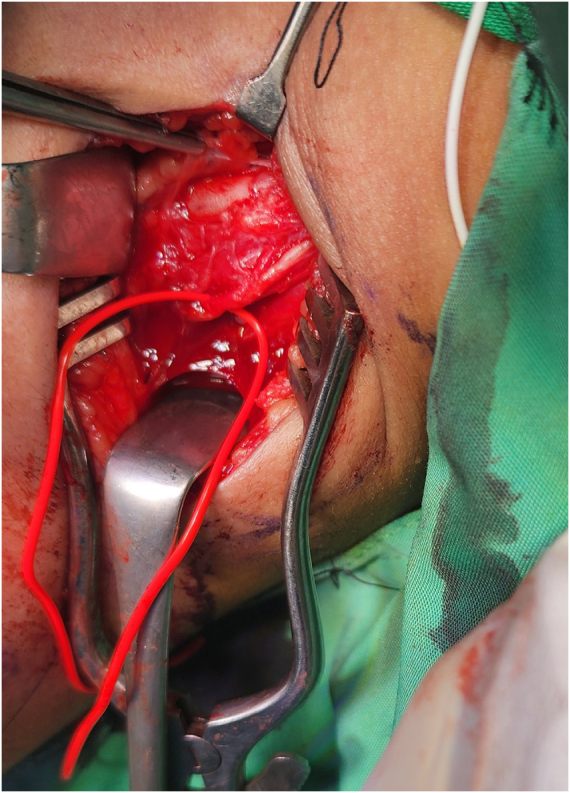
Suprascapular nerve after excision of bony envelope.

The first stage of surgery was done 3 months after the onset of symptoms. The patient underwent general anesthesia. A supraclavicular incision was made, and the brachial plexus was identified. The nerve sheath appeared calcified (Fig. 2). The calcification appeared as a bony envelope encasing the nerve, which was easily detached with scissors (Fig. 3) and subsequently excised (Fig. 4). A sample was taken for histological examination. The nerve was severely damaged, and a neuroma was found. All trunks of the brachial plexus were non-stimulable.

Based on the intraoperative finding, nerve transfers were performed. The phrenic nerve was transferred to the suprascapular nerve, and the spinal accessory nerve was transferred to the main trunk of the musculocutaneous nerve using a medial antebrachial cutaneous nerve graft. An amniotic membrane wrap was applied to the nerve anastomosis. The wound was sutured, and the first stage of the surgery was done. Postoperatively, the patient’s arm was put in an arm sling.

One month postoperatively, the patient presented to the orthopedic clinic for a follow-up. On physical examination, she was able to abduct her shoulder at 2/5, while biceps, brachialis, and hand function remained absent. The histopathological examination of the intraoperative sample revealed cartilaginous tissue without evidence of malignancy. She was subsequently scheduled for the second-stage surgery for the median nerve.

The second-stage surgery was done 5 months after the onset of symptoms. The aim of the second-stage surgery was to restore median nerve function. This was done by transferring the C5 nerve root to the median nerve using a pedicled ulnar graft.

One month after the second-stage surgery, the physical examination showed that the patient was able to abduct her shoulder at 2/5, with no recovery of elbow flexion and hand function. She was referred to the physical medicine and rehabilitation clinic for electrical stimulation.

Four months after the second-stage surgery, physical examination of her left arm showed: shoulder shrug 5/5, shoulder abduction 3/5, elbow flexion and extension 0/5, wrist flexion and extension 0/5, finger flexion and extension 0/5, and thumb extension 0/5.

**Figure 5.**
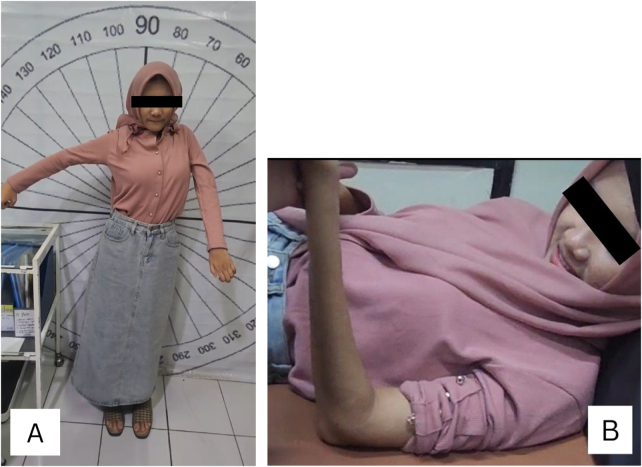
Clinical evaluation at 1-year follow-up. (A) Active shoulder abduction to 40°. (B) Elbow flexion at 2/5.

One year after the second-stage surgery, the patient demonstrated shoulder flexion at 2/5, active shoulder abduction to 40° at 3/5, elbow flexion at 2/5, elbow extension at 4/5, wrist flexion at 2/5 and extension at 0/5, finger flexion at 2/5 and extension at 0/5, and thumb extension at 0/5 (Fig. 5). She was subsequently referred to the physical medicine and rehabilitation clinic.

## Discussion

Neuritis ossificans is a rare disorder that is characterized by heterotopic ossification, or abnormal bony tissue formation, within a nerve^[^1,2^]^. It occurs more commonly in the upper than the lower extremities[6]. Past studies have reported cases of neuritis ossificans occurring spontaneously or after a trauma. Clinical manifestations include pain, weakness, and numbness in the affected nerve distribution^[^4,6–13^]^. Neuritis ossificans has been reported in the brachial plexus^[^3,4^]^, cranial nerve[7], median nerve[9], common peroneal nerve[12], ulnar nerve[10], radial nerve[8], and sciatic nerve^[^6,13^]^.

Neuritis ossificans primarily involves the epineurium. The pathophysiology remains unclear. However, recent theory hypothesizes that neuritis ossificans shares a similar pathophysiology as heterotopic ossification of the muscles that occurs after a trauma^[^6,14^]^. In heterotopic ossification involving the nerve, there is a disruption in the blood-nerve barrier that is accompanied by the release of bone morphogenetic protein-2 from the bone and soft tissues around the affected nerve.

Furthermore, during nerve inflammation, mast cell degranulation releases chemoattractants for platelets, neutrophils, macrophages, which further compromise the blood–nerve barrier. The damage to this barrier allows perineural fibroblasts and osteoprogenitor cells to infiltrate the endoneurium and continue to migrate toward the epineurium. The bone morphogenetic proteins (BMP) that were released subsequently promote mesenchymal stem cell differentiation and fibroblast conversion toward osteoblasts, thereby triggering heterotopic ossification^[^4,7^]^.

The symptoms of neuritis ossificans arise from compression of the affected nerve and surrounding soft tissues by the ossification. The patient typically presents with pain along the sensory distribution of the affected nerve. Nerve compression may also result in muscle weakness in the corresponding innervated muscles. On physical examination, a palpable calcified mass along the course of the nerve may be felt[1].

Ancillary investigations such as CT scan, ultrasound, or MRI may reveal a mass of calcification along the course of the affected nerve. MRI is considered the most reliable imaging method to assess perineural edema and inflammation. Ultrasound can reveal an echogenic mass of ossification, although nerve structure may not be visualized due to acoustic shadowing[9]. In our case, we performed preoperative shoulder CT scan and plain radiography examination, as the patient presented with post-traumatic arm paralysis, raising initial suspicion of brachial plexus injury with or without an associated fracture, rather than neuritis ossificans. The preoperative axial shoulder CT scan demonstrated an ossified mass located posterior to the clavicle. We propose the term *double clavicle* sign to describe this finding, defined as a bone-density mass representing ossification of the brachial plexus situated between the clavicle and the first rib. The electromyography and nerve conduction velocity study concluded a total functional lesion of the left C5 nerve root and preganglionic denervation of the rest of the left brachial plexus.

Histologically, neuritis ossificans consists of three distinct regions: a central zone of fibrovascular tissue, an intermediate zone, and a peripheral zone of lamellar bone[9]. Histopathological examination is essential to identify the origin of the tissue. The differential diagnosis includes non-neural heterotopic ossification with secondary nerve involvement, ossification secondary to metabolic disorder of calcium and phosphate, and nerve calcification secondary to diabetes^[^6,11^]^.

The optimal treatment for neuritis ossificans remains unknown due to its rarity. A few studies have reported favorable outcomes with surgical excision^[^1,8^]^. Pharmacological management may include nonsteroidal anti-inflammatory drugs and GABA inhibitors for neuralgia^[^4,12^]^. Physiotherapy has also been reported to reduce pain[12].In our case, surgical intervention was performed in two stages, followed by physiotherapy and electrical stimulation, which led to gradual neurological improvement.

To date, only two cases of neuritis ossificans of the brachial plexus have been reported. In 1946, Woltman et al. reported a case of post-traumatic brachial plexus neuritis ossificans. The patient initially showed partial neurological recovery over 3 years, but her condition deteriorated during the following 4 years. During brachial plexus exploration, the brachial plexus was found to be calcified, with the center of the nerve appearing to be softer than the periphery. The lesion was thought to be a result of degeneration followed by ossification. However, the pathology examination report suggested that the tissue was a grade I osteogenic sarcoma that was differentiating into a mature bone[3]. However, 64 years later, Spinner et al. re-examined the original specimen and concluded that the osteosarcoma was not related to the neuritis ossificans[15]. In contrast, the pathology examination in our case revealed cartilaginous tissue without evidence of malignancy.

The second reported case of neuritis ossificans of the brachial plexus was in 2020, by Murthy et al. Unlike the earlier reported case, this patient had no history of trauma despite being physically active. The patient presented with progressive neurological deterioration. The diagnosis of neuritis ossificans was confirmed through MRI and histopathological examination. The patient was managed conservatively with indomethacin (75 mg daily for 6 weeks), followed by naproxen nightly. Two months later, his symptoms improved[4].

We report the first case of neuritis ossificans of the brachial plexus following a total brachial plexus injury. In our case, pharmacological treatment was unlikely to be effective due to complete denervation of the brachial plexus. Therefore, surgical management was undertaken, consisting of excision of the calcification and nerve transfers to the denervated brachial plexus. The outcome was satisfactory, with visible improvement within 2 months after the first stage of the surgery.

## Conclusion

Neuritis ossificans is a rare disorder thought to share similar pathophysiology with myositis ossificans. We present the first case of neuritis ossificans of the brachial plexus after a total brachial plexus injury. We propose the term *double clavicle* sign to describe a bone-density mass representing ossification of the brachial plexus between the clavicle and the first rib visible in axial CT images of the shoulder. We reported that surgical excision of the ossification and nerve transfer of the denervated brachial plexus yielded a satisfactory outcome.

## Data Availability

The data that support the findings of this study are available from the corresponding author upon reasonable request.

## References

[1] Isla A Pérez-López C De Agustín D. Neuritis ossificans of the sciatic nerve: case illustration. J Neurosurg 2004;101:545. 15352617 10.3171/jns.2004.101.3.0545

[2] Meyers C Lisiecki J Miller S. Heterotopic ossification: a comprehensive review. JBMR Plus 2019;3:e10172. 31044187 10.1002/jbm4.10172 PMC6478587

[3] Woltman HW Adson AW. Neuritis ossificans with osteogenic sarcoma in brachial plexus following trauma; report of case. J Lancet 1946;66:372–76. 21001663

[4] Murthy NK Fritchie KJ Amrami KK. Diffuse neuritis ossificans of the brachial plexus: case report and review of the literature. World Neurosurg 2020;141:363–66. 32599197 10.1016/j.wneu.2020.06.154

[5] Kerwan A Al-Jabir A Mathew G. Revised Surgical CAse REport (SCARE) guideline: an update for the age ofArtificial Intelligence. Prem J Sci 2025;10:100079.

[6] Yuga ACQ Pascual JSG Valparaiso AP. Extensive neuritis ossificans of the sciatic nerve: systematic review and illustrative case. J Clin Neurosci 2022;98:224–28. 35202993 10.1016/j.jocn.2022.02.021

[7] Kemper CM Rojas JC Bauserman S. Neuritis ossificans of a cranial nerve. J Neurosurg 2010;113:1112–14. 20509727 10.3171/2010.5.JNS091931

[8] Muzaffar N Ahmad N Bhat A. Neuritis ossificans of the radial nerve. Orthopedics 2012;35:e589–9. 22495866 10.3928/01477447-20120327-39

[9] Lee KH Jeong YM Jeon JY. Sonographic diagnosis of neuritis ossificans of the median nerve. J Clin Ultrasound 2018;46:358–60. 29044622 10.1002/jcu.22544

[10] Sammons M Tami I Giesen T. Neuritis ossificans of the ulnar nerve at the elbow: a case report. J Hand Surg Eur Vol 2021;46:783–84. 33726548 10.1177/1753193421999777

[11] Choi KH Park SG Baek JH. Myositis ossificans causing ulnar neuropathy: a case report. J Int Med Res 2021;49:03000605211002680. 33771066 10.1177/03000605211002680 PMC8166390

[12] Trigkilidas D Lidder S Delaney D. Neuritis ossificans of the common peroneal nerve: a case report. Skeletal Radiol 2009;38:1115–18. 19669757 10.1007/s00256-009-0766-y

[13] Bellasri S El Asri C. Neuritis ossificans: rare cause of sciatica. Pan Afr Med J 2016;25:1–2. 10.11604/pamj.2016.25.170.9937 PMC5326069 28292132

[14] Wasman JK Willis J Makley J. Myositis ossificans-like lesion of nerve. Histopathology 1997;30:75–78. 9023561 10.1046/j.1365-2559.1997.d01-555.x

[15] Spinner RJ Scheithauer BW. Neuritis ossificans of the common peroneal nerve. Skeletal Radiol 2010;39:311–12. 20037796 10.1007/s00256-009-0851-2

